# Postdischarge Environment Following Heart Failure Hospitalization: Expanding the View of Hospital Readmission

**DOI:** 10.1161/JAHA.113.000116

**Published:** 2013-04-24

**Authors:** Andrew M. Hersh, Frederick A. Masoudi, Larry A. Allen

**Affiliations:** 1 Department of Internal Medicine, University of California, Davis, CA (A.M.H.); 2 Department of Internal Medicine, David Grant USAF Medical Center, Travis AFB, CA (A.M.H.); 3 Colorado Cardiovascular Outcomes Research Consortium, Denver, CO (F.A.M., L.A.A.); 4 Division of Cardiology, University of Colorado Anschutz Medical Campus, Aurora, CO (F.A.M., L.A.A.)

**Keywords:** heart failure, patient readmission, quality improvement

## Introduction

Readmission after hospitalization for heart failure (HF) has received increasing attention due to the significant burden it places on patients and payers.^1–2^ Among Medicare beneficiaries, readmission within 30 days following heart failure hospitalization approaches 25%.^2^ Even after adjusting for case mix, significant variation in hospital readmission rates exists. This hospital‐level variation suggests that many of these readmissions may be preventable.^3^ HF readmission rates adjusted for risk using a claims‐based model are now publicly reported as a measure of institutional quality (www.HospitalCompare.hhs.gov). As of October 2012, the Patient Protection and Affordable Care Act's (PPACA) value‐based purchasing policies began reducing Medicare payments to hospitals with “excess” HF readmissions and offered new funding opportunities for innovative approaches to reduce HF readmissions.^4^

Despite the obvious value of reducing unnecessary readmissions, the way forward is not as clear as these policies might suggest. An increasing segment of the medical community is voicing concern with the extent to which public reporting and financial penalties positively influence institutional HF readmission rates.^5^ Value‐based purchasing may unfairly punish hospitals that provide care to socioeconomically disadvantaged patients and incentivize the avoidance of high‐risk patients^6–9^ due to perceived inadequacies of current risk standardization models.^10^ In addition, effective interventions to prevent unnecessary readmissions remain elusive.^11^

Prior efforts to identify risk factors for HF readmission have put an inordinate priority on the convenience of data collection. The vast majority of existing risk models employ administrative billing and inpatient clinical data from a single episode of care that are not designed to fully elucidate the breadth of potential causes of readmission. Notably missing are factors reflecting the patient's postdischarge environment. Recent literature suggests that “social instability”—a term which reflects a relative lack of social support, education, economic stability, access to care, and safety in the patient's environment—is an important mediator of readmission risk.^12–13^

Within this context, we set out to (1) review what is known about the postdischarge environment and its relationship to HF readmission, and (2) propose a new conceptual model for HF readmission that integrates patient, provider, health system, and environmental factors. Doing so has the potential to improve the predictive capacity of HF readmission risk models, thereby making quality measures fairer, and to guide us in improving transitions of care, and ultimately leading toward reductions in unnecessary readmissions.

## Literature Search

The concept of the postdischarge environment has not been a clearly defined domain in current readmission literature. Therefore, the approach taken was to systematically identify all readmission models and then manually extract factors that were perceived to represent the postdischarge environment. Systematic reviews of the literature regarding HF readmission risk models have been performed previously by Kansagara et al^10^ (2011) and Ross et al^14^ (2008). We used the published Kansagara search alogrithm to capture newer literature published up to November 15, 2012. In addition, we supplemented the Kansagara search algorithm with an additional search focusing specifically on the postdischarge environment using the terms “postdischarge environment, environment, social, social instability, education, poverty, economic, and socioeconomic” in combination with “readmission and/or rehospitalization” and any medical or surgical condition. We then reviewed abstracts and included studies which explored the relationship of readmission to one or more aspects of the postdischarge environment.

Models identified from these searches that included any factor representing the postdischarge environment are summarized in the Table.

**Table 1.** Selected Heart Failure and General Readmission Risk Models Focusing on Patient, System, and Environmental Level Covariates

Year	Model	Patient					System		Environment	
		Demographic Covariates	Indicator of Frailty or Functional Status	Comorbidities	Markers of Illness Severity	Use Patterns	Hospital Characteristics and Postdischarge Services	Readiness for Discharge or Inpatient Quality	Finances, Education, Stability, and Support	Patient Behavior
**1985**	Predicting hospital readmissions in the Medicare population^15^	**Age, sex, race**	Disability status	None	None	**No. of discharges in previous 60 days**, no. of discharges with same dx in past 60 days, LOS, **hospital reimbursement**,** admission for chronic vs acute diagnosis**	**Hospital based characteristics**, region of the country	None	**Disability status, supplemental Medicaid coverage**	None
**1988**	Identifying factors associated with health care use: A hospital‐based risk screening index^16^	**Age > 75**	**Dependent ambulation**,** incontinence, poor mental status**, terminal illness	**2+ chronic condition**, terminal illness, **psychiatric disease**	None	Emergency admission, prior hospitalization within the past 2 months	None	None	**Unmarried**,** less than subsistence level income**,** lives alone or in SNF**, dependent self care (requires help with ADLs), unemployment or receiving disability, poor social support	History of alcoholism
**1988**	Postdischarge care and readmission^17^	Age, sex, race	None	None	**BUN, paO2, WBC, hemoglobin**	**ER visits in previous 6 months**	**Post discharge care including RN calls, mailings, and appointment reminder vs usual care**	None	None	None
**1990**	Risk Factors for early readmission among veterans^18^	**Age,** sex, race, period of military service, county of residence	None	Spinal cord injury, **number of surgeries performed**,** risk category for admitting diagnosis**	None	**LOS, unit type (medical, intermediate, neurological, surgical),** discharged against medical advice	VA auspices, place and type of disposition	None	**Compensation/pension status, distance from hospital services**, marital status, former POW	None
**1991**	Factors predicting readmission of older general medicine patients^19^	Age, race	Cognition (MMSE)	Depression, **diagnoses at admission (CHF or COPD)**	Illness severity (Computerized Severity of Illness Index)	**Emergent hospitalization**,** no. of hospitalizations in the last year**,** no. of days hospitalized in the last year**, LOS, admitted from home	None	None	Income, level of education, marital status, lives alone, meeting ADLs	Novne
**1992**	Contribution of a measure of disease complexity (COMPLEX) to prediction of outcome and charges among hospitalized patients^20^	Age, sex	None	Used a metric comprised of a count of significantly effected body systems as well as a comorbidity severity score	None	None	None	None	None	None
**1996**	Does risk‐adjusted readmission rate provide valid information on hospital quality^19^	**Age, sex**	None	“complexity” measured as number of PMCs present	**PMC Relative Intensity Score**	**LOS**	None	None	None	None
**1997**	Correlates of early hospital readmission or death in patients with Congestive Heart Failure^21^	Age, sex, race	None	History of MI, HF, VT/VF, DM, **Charlson Comorbidity Index**	EF, **systolic BP**, respiratory rate, serum sodium, serum creatinine, cardiomegaly on admission CXR, NSR on admission EKG, **absence of new ST‐T changes on admission EKG**	None	Has a PCP	Symptoms at discharge, laboratory abnormalities at discharge	Income, education, **single**, person at home to help with medical care	None
**1999**	Prediction of hospital readmission for heart failure: development of a simple risk score on administrative data^22^	Age, sex, **race**	None	Charlson Comorbidity Index, **specific comorbid conditions**	See use pattern	LOS, total hospital discharge dollars, use of an ICU, procedural complication, **discharge to SNF**, transfer to acute care hospital, **home health services after discharge**, discharged AMA, Cardiology service, PT/OT, specific noninvasive cardiology procedures **(echo**,** telemetry monitoring**, EST, etc.), invasive cardiac procedures (PCI, CABG, etc.), critical care procedures (pulmonary artery catheterization, inotropic agents, mechanic ventilator support, HD, etc.)	**Hospital location, hospital type**	None	**Insurance type**	History of drug or alcohol abuse
**2000**	Predicting nonelective hospital readmissions: A multi‐site study^23^	Age, gender, race	SF‐36 score physical component summary	**SF‐36 mental component summary**	Disease specific severity markers (eg, insulin dependence, home O_2_ use, NYHA class), discharge lab values including **BUN**, Hb, WBC	**No. of ED visits in previous 6 months**, no. **of admissions in previous 6 months**, LOS, **patient satisfaction scores from survey data**	None	None	Marital status, highest grade completed, distance from VAMC, employment status, service connection	None
**2000**	Predictors of readmission among elderly survivors of admission with heart failure^24^	Age, sex, race	Discharge mobility	**Specific comorbid conditions**	Presences of PND, orthopnea, chest pain, systolic/diastolic blood pressure, respiratory rate, pulmonary edema on CXR, LVEF, occurrence during hospitalization of a major complication (cardiac arrest, shock, MI, stroke), major procedure during hospitalization (CABG, cardiac catheterization), labs at discharge including: sodium, **BUN**, creatinine, BUN/CR ratio, ACE inhibitor prescription, digoxin prescription	**Previous admission within 1 year**, LOS	None	None	None	None
**2004**	Posthospital care transitions: patterns, complications, and risk identification^25^	**Age, sex**	**Premorbid functional status score**,** self‐rated general health**,** visual impairmen**t, **need for assistance with ADLs**	**Charlson Comorbidity Index**,** specific comorbid conditions**,** Alzheimer's disease**	None	**Previous admission and average LOS in the previous 6 months**,** number of prior SNF stays and average LOS in previous 6 months**	None	None	**Medicaid status**,** unmarried**	None
**2004**	Risk stratification after hospitalization for decompensated Heart Failure^26^	Age, gender, race	None	Specific comorbid conditions	Duration of HF diagnosis, HF etiology, **history of PCI**, presence of peripheral edema, S3 murmur, EF, NYHA class, JVD, HJR, rales, heart rate, **systolic BP**, diastolic BP, respiratory rate, K, **BUN**, Cr, Na, platelets, **Hb**	**Number of prior HF hospitalizations in the previous 12 months**	None	None	None	None
**2006**	Identifying patients at high risk of emergency hospital admissions: A logistic regression analysis^27^	**Age**,** sex, ethnicity, Std admission ratio**	None	**Charleson Comorbidity Index**,** presence of an “ambulatory care sensitive condition”**	None	**No. of ED visits in past 365 days**,** no. of ED visits in the past 366 days to 36 months**, number of consultant episodes in “index spell”	None	None	**Area‐level lifestyle group**,** area‐level deprivation**,** source of admission**	None
**2006**	Validation of the potentially avoidable hospital readmission rate as a routine indicator of the quality of hospital care^28^	**Age, sex**	None	**Charlson Comorbidity Index**,** number of comorbidities**,** diagnoses at admission**	None	**Previous admission in the past 6 months**	None	None	None	None
**2007**	Improving the management of care for high‐cost Medicaid patients^29^	Age, sex, race/ethnicity	None	**Number and type of comorbidities**,** history of mental illness**,** history of alcohol or substance abuse**	None	**Frequency and interval of hospitalizations, ED visits**,** primary care visits, and specialist care visits in previous 3 years**,** Use of home health care, personal care, rehab services**,** substance abuse services**,** prescription medications**,** inpatient spending**	None	None	**Socioeconomic status of the zip code of residence**	None
**2007**	Prediction of Rehospitalization and Death in Severe Heart Failure by Physicians and Nurses of the ESCAPE Trial^30^	**Age, sex, race**	**6 minute walk distance**	None	**NYHA class**,** need for “high dose” loop diuretic**,** ischemic vs nonischemic HF**,** systolic BP**,** diastolic BP, HR, NA, Cr, BUN, EF, required CPR, required mechanical ventilation**	None	None	**Beta blocker prescribed at discharge, ACE inhibitor prescribed at discharge**	None	None
**2008**	Hospital 30‐day Heart Failure readmission measure: methodology^31^	**Age, sex**	**Protein calorie malnutrition**	**Specific comorbid conditions**	None	None	None	None	None	**Drug or alcohol abuse**
**2008**	Risk factors for 30‐day hospital readmission in patients ≥ 65 years of age^32^	**Age, sex, race/ethnicity**	None	**Specific comorbid conditions**	None	Service type (medical vs surgical)	**Discharge destination**	None	Insurance status, distance from hospital, median income of zip code of residence	None
**2009**	Using routine inpatient data to identify patients at risk of hospital readmission^33^	**Age,** sex, indigenous status,	None	**Specific comorbid conditions**	None	**Previous admission in the preceding 90 days, 1 year**,** or 3 years**, previous emergency admission in the preceding 90 days, 1 year, and 3 years	None	None	Marital status, **socioeconomic status**, rurality, **geographic remoteness**	None
**2010**	An automated model to identify heart failure patients at risk for 30‐day readmission or death using electronic medical record data^13^	Age, **sex,** race	See markers of severity	**Depression or anxiety**	**Tabak Mortality Risk Score** (derived from albumin, total bilirubin, CK, creatinine, sodium, BUN, Pco_2_, WBC, troponin‐I, glucose, INR, BNP, ph, temperature, pulse, diastolic BP, systolic BP)	**Number of prior admissions**,** ED visits, and outpatient visits, presentation to ED from 6 am** **to 6 pm**	None	None	**Socioeconomic status**,** single**,** payment method**,** use of a health system pharmacy**	**Cocaine use**, history of leaving AMA, **missed outpatient appointments, number of home address changes**
**2010**	Derivation and validation on an index to predict early death or unplanned readmission after discharge from hospital to the community^34^	Age, sex	Dependent for one or more ADL	**Charlson Comorbidity Index**	None	**LOS, visit to the ED in the previous 6 months**, hospital admissions within the previous 6 months, medical vs surgical admission, **emergent admission**, number of medications at discharge, number of new medications at discharge, season at discharge, consultation, number of complications while hospitalized	Has a PCP	None	Lives alone	None
**2010**	Hospital readmission in general medicine patients: A prediction model^35^	Age, sex, race/ethnicity	**SF‐12 physical component**, MMSE, presence of functional limitation	**Charlson Comorbidity Index**, SF‐12 mental component	None	**Number of admissions in the previous year**,** LOS,** need for extra day stay during current admission	**Has a PCP**	None	Household income, education, **primary insurance**,** marital status**, lives alone, someone available to help with care	None
**2011**	Inability of providers to predict unplanned readmissions^36^	Age, sex	Poor self‐rated general health	CAD, DM2	None	Admission in prior year, more than 6 doctor visits in prior year	None	None	None	None
**2011**	Incremental value of clinical data beyond claims data in predicting 30‐day outcomes after heart failure hospitalization^37^	**Age**,** sex**	None	**Diagnoses at admission including psychiatric diagnoses**	EF, heart rate, **hemoglobin, serum creatinine, serum sodium**, systolic **blood pressure**, weight	None	None	None	None	None
**2011**	Unplanned readmissions after hospital discharge among patients identified as being at high risk for readmission using a validated predictive algorithm^38^	None	None	**Charlson Comorbidity Index**	None	**LOS**,** number of ED in the previous 6 months**,** emergent admission**	None	None	None	None

Bolded covariates were included in the final model. Nonbolded covariates were proposed, but not included. LOS indicates length of stay; SNF, skilled nursing facility; ADL, activities of daily living; WBC, white blood cell; MMSE, mini‐mental state examination; CHF, congestive heart failure; HF, heart failure; VT, ventricular tachycardia; EF, ejection fraction; PCP, primary care provider; EST, exercise stress test; PCI, percutaneous coronary intervention; CABG, coronary artery bypass graft; HD, hemodialysis; Hb, hemoglobin; LVEF, left ventricular ejection fraction; ACE, angiotensin‐converting enzyme; CK, creatine kinase; INR, International Normalized Ratio; BNP, brain natriuretic peptide; CAD, coronary artery disease.BUN, blood urea nitrogen; RN, registered nurse, VA, Veteran's Administration; POW, prisoner of war; COPD, chronic obstructive pulmonary disease; PMC, patient management category; MI, myocardial infarction; VF, ventricular fibrillation; DM, diabetes mellitus; BP, blood pressure; CXR, chest x‐ray; NSR, normal sinus rhythm; EKG, electrocardiogram; ST‐T, ST or T segment; ICU, intensive care unit; PT/OT, physical therapy/occupational therapy; SF‐36, Short Form‐36; NYHA, New York Heart Association; ED, emergency department; VAMC, Veteran's Affairs Medical Center; PND, paroxysmal nocturnal dyspnea; CR, creatinine; JVD, jugular venous distention; HJR, hepatojugular reflux; ESCAPE, Evaluation Study of Congestive Heart Failure and Pulmonary Artery Catheterization Effectiveness; HR, heart rate; NA, sodium; CPR, cardiopulmonary resuscitation.

Using these results we then attempted to synthesize the information into a conceptual model of HF readmission (Figure), paying special attention to postdischarge environmental factors.

**Figure 1.**
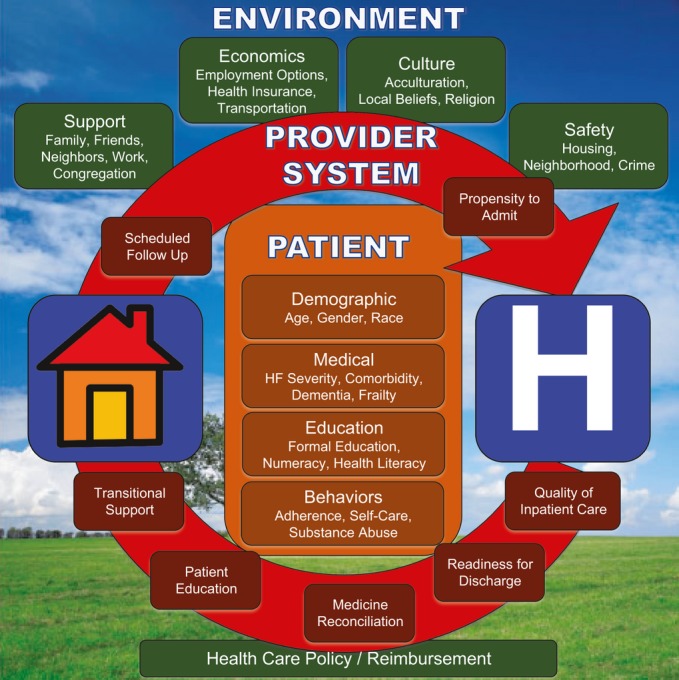
Proposed conceptual model for heart failure readmission, emphasizing that the patient and health care provider work within their environment.

## The State of Heart Failure Readmission Risk Modeling

Prediction models play a vital role in our understanding, interpretation, and reaction to HF readmissions. They provide insight into the primary factors that underlie readmission, and as such, point toward new and more focused interventions. Furthermore, an understanding of individual patient risk allows hospitals to triage costly, high‐intensity interventions to those patients most likely to derive benefit from them. Finally, readmission rates, adjusted for patient factors, have been used to measure institutional quality of care. Thus appropriate risk modeling is vital for creating “apples‐to‐apples” comparisons between different institutions, as well as within a single institution over time.

Risk factors and associated prediction models for HF readmission have been systematically described elsewhere.^10,14^ Although several HF readmission risk models have been validated and published, the state of risk prediction in HF readmission remains crude. The ability to discriminate patients who will be readmitted from those who will not is significantly lower than it is for postdischarge mortality, with C‐indices for HF readmission models rarely exceeding 0.70.^13,22,24,26,30,37^ Likewise the ability of providers to predict HF readmission via “clinical gestalt” appears similarly limited.^36^ The reasons are multiple. First, a relatively high proportion of readmissions may be inherently stochastic events, and therefore, models of readmission will have some “ceiling” of predictive performance. Second, variation in readmission risk following adjustment for patient‐level factors may be partially attributable to provider and system‐level differences in care delivery (ie, differences in quality). Third, existing models might fail to reliably predict some readmissions because they are missing key domains that drive its occurrence.

Existing prediction models have relied heavily on data collected during hospitalization, typically from inpatient clinical registries and claims‐based administrative data (Table). This “data first” approach uses readily available data to dictate the hypotheses to be tested, rather than the other way around. It largely neglects some difficult‐to‐measure, but logical, domains. These include complex comorbid disease, frailty, subclinical depression and anxiety, substance abuse, cognitive limitations, lack of formal and informal education (health literacy, numeracy), acculturation, suboptimal patient adherence, inability to provide self‐care, caregiver support, and social networks.

## The Importance of the Postdischarge Environment

Although easily captured measures of a patient's postdischarge environment have been considered in some existing models (eg, income, marital status, insurance status; Table), a systematic approach to this domain has been largely absent from the HF readmission discussion. New data are emerging to indicate that stability in the postdischarge environment plays a critical part in HF readmission.

Amarasingham et al derived and validated an HF readmission risk model within a large, inner city, safety‐net hospital, using a wide range of automated data gleaned from the electronic medical record.^13^ In this multivariable analysis, several factors emerged that were associated with 30‐day readmission, including being single, male, using Medicaid, having an increased number of address changes, average income level for zip code of residence, and time of presentation to the ED (between 6 am and 6 pm). When these markers of “social instability” were included as a group into a previously validated model, the 30‐day risk prediction improved markedly (C‐statistic from 0.61 to 0.72). This suggests that social environmental factors are important determinants of readmission risk.

A second study by Arbaje et al further supports this hypothesis.^12^ Using Medicare claims data as well as the Current Beneficiary Survey, this group looked at the relationship between socioeconomics, the postdischarge environment, and the likelihood of early hospital readmission over a range of diseases, including HF. In the study's population, being unmarried, living alone, lacking “self‐management skills”, and having an unmet activity of daily living and lower level of education put a patient at increased risk for readmission. Interestingly, after adjusting for these other factors, no direct relationship was found between income and risk.

A variety of studies have shown that indigent populations tend to have higher rates of HF readmission. An analysis of national Medicare data showed that 30‐day HF readmission rates for Medicare beneficiaries were higher among black patients than white patients, and that patients from minority‐serving hospitals had higher readmission rates than those from nonminority‐serving hospitals.^39^ Even after adjustment for measured clinical factors, Medicaid populations had higher HF readmission rates than their commercially insured counterparts.^40^ Some portion of these differences may be due to inferior health care for these populations, but differences in patient and environmental factors not captured by existing models are likely to contribute as well. At least among the Medicare population, community measures explain far more of the variance in institutional HF readmission rates than do hospital process performance measures.^41^

Recent analyses that have specifically collected data on social factors not captured by traditional databases (a “hypothesis first” approach) have helped expand our view of the mediators of readmission. Peterson et al showed in a series of papers derived from prospective health survey information that health literacy^42^ and acculturation^43^ were strong predictors of adverse outcomes after discharge among patients hospitalized with HF, and Tao et al^44^ suggest a scoring system that might be used to predict patients whose social situation place them at higher risk for readmission.

As further evidence of the influence of the postdischarge environment on readmission, successful interventions that have effectively reduced readmissions have generally done so by altering the patient's postdischarge environment or the patient's ability to manage his/her own environment. For example, comprehensive discharge planning (including education of the patient and family), social‐service consultation, and intensive follow‐up were components of the earliest successful HF readmission interventions.^45–46^ More recently, transition coaches who go directly into the home environment to support a variety of patient needs have been shown to be effective.^47^ Unlike successful interventions that use trained personnel to broadly support patients in their transition to home, unimodal interventions^11^ and those focused primarily on the physiology of HF^48^ have consistently failed to reduce HF readmission rates.

## A New Conceptual Model for HF Readmission

HF readmission is an event that occurs, by definition, in the postdischarge environment. As such, it is reasonable to surmise then that this environment would act as a mediator. Based on our current understanding of readmissions, we propose a new explicit paradigm of HF readmission that positions patient and health system factors within their relevant environment (Figure). The patient interacts with the provider and health system all within the context of the surrounding environment. This conceptualization moves the postdischarge environment from a peripheral (or ignored) role to an encompassing one. Changing our conceptualization transforms our view of readmission from a biological, hospital‐based event to a “sociobiological” process. This new model also helps reconcile how patient factors and provider/health system factors relate to each other through the postdischarge environment. Concretely, this reframing suggests how new lines of research into the postdischarge environment may lead to further improvements in our ability to predict and mitigate risk of readmission.

The question of how the postdischarge environment affects readmissions is important. Readmission is typically a multifactorial process.^49^ We hypothesize that increased stability in the postdischarge environment can positively affect a variety of domains related to readmission. Social stability has the potential to improve dietary compliance and fluid restriction, increase medication adherence, increase access to health care and improve compliance with appointments, raise levels of exercise, reduce tobacco and alcohol use, etc. Together, these factors may positively influence HF severity and disease progression. In addition, they may decrease comorbidity number and severity and even help bolster a patient's physiologic reserve. These domains may remove barriers to, or combine with, provider and systems‐based factors to synergistically influence rates of readmission.

## Environmental Factors and Public Policy

It has been has been argued that socioeconomic factors have a limited place in risk modeling because adjusting for them may “excuse” substandard care for indigent and impoverished populations.^5–9,50^ To the contrary, acknowledging that the patient and health system reside within a larger environment counters this argument. Including environmental factors in risk‐standardization models for public reporting and value‐based purchasing recognizes the unique challenges posed by patients with significant environmental instability. In addition, this perspective lends support to incentives that would foster the development of innovative transitional care programs in order to accommodate social instability or directly enhance the patient's ability to navigate the postdischarge environment. Moving from the overly simplistic, dichotomous, patient‐hospital construct to consideration of the patient, clinician, and hospital as members of the community in which they all reside promotes a more integrated approach to health. Ultimately, major improvements in the health of patients with chronic, progressive diseases (like HF) will require coordinated efforts among patients, families, providers, health systems, governmental agencies, and community organizations. This integrated approach should be properly incentivized by sound public policy.

As the Centers for Medicare and Medicaid Services scale up performance‐based payments, it must consider the potential influence of socioeconomic factors on outcomes to ensure that hospital payment penalties do not exacerbate disparities in care. Although outcome measures designed to reduce unnecessary hospital readmissions may be an important step forward in advancing quality in some respects, the failure to incorporate environmental factors could influence hospitals' ability and willingness to serve vulnerable populations.^51^ Stratifying institutional readmission results by important environmental factors may be one way to “level the playing field” when assessing hospital performance and encourage hospitals to maintain access to care for vulnerable populations.

## Future Research

Factors related to the postdischarge environment need to be better explored, measured, and integrated into risk models and interventions. Without a comprehensive and systematic analysis of the postdischarge environment, we are unlikely to realize reductions in unnecessary HF readmissions. Such an approach would involve a number of steps, including the development of definitions and an associated taxonomy around relevant factors in the postdischarge environment followed by surveillance of these factors through an explicit mechanism.^52^

Research by Ross et al^14^ and Arbaje et al^12^ provides an example of how to assess the incremental value of “factors of social instability” by assessing risk model performance before and after inclusion of these factors. In the meantime, institutions that are seriously working to improve their HF readmission rates should recognize that interventions that ignore the environment into which a patient is discharged are unlikely to significantly impact their readmission rates.

## Conclusions

A variety of forces, including passage of the PPACA and its linkage of HF readmission to reimbursement, have placed HF readmissions at the forefront of quality improvement efforts in medicine. However, the poor performance of existing HF readmission risk models combined with our failure to significantly impact HF readmission rates^53^ should give us pause. HF readmission consists of a complex interplay between patient, health system, and the environment. We believe that conceptualizing HF readmission as a sociobiological process rather than a discrete physiologic occurrence will help us to better characterize, predict, and ultimately mitigate risk. Further research into the exact mechanisms by which the postdischarge environment affects readmission will improve quality measures and future interventions designed to keep HF patients out of the hospital.
